# Reconciling species diversity in a tropical plant clade (*Canarium*, Burseraceae)

**DOI:** 10.1371/journal.pone.0198882

**Published:** 2018-06-15

**Authors:** Sarah Federman, Michael J. Donoghue, Douglas C. Daly, Deren A. R. Eaton

**Affiliations:** 1 Department of Ecology and Evolutionary Biology, Yale University, New Haven, CT, United States of America; 2 Institute of Systematic Botany, New York Botanical Garden, Bronx, NY, United States of America; 3 Department of Ecology, Evolution, and Environmental Biology, Columbia University, New York, NY, United States of America; Vanderbilt University, UNITED STATES

## Abstract

The challenges associated with sampling rare species or populations can limit our ability to make accurate and informed estimates of biodiversity for clades or ecosystems. This may be particularly true for tropical trees, which tend to be poorly sampled, and are thought to harbor extensive cryptic diversity. Here, we integrate genomics, morphology, and geography to estimate the number of species in a clade of dioecious tropical trees (*Canarium* L.; Burseraceae) endemic to Madagascar, for which previous taxonomic treatments have recognized between one and 33 species. By sampling genomic data from even a limited number of individuals per taxon, we were able to clearly reject both previous hypotheses, and support instead an intermediate number of taxa. We recognize at least six distinct clades based on genetic structure and species delimitation analyses that correspond clearly with geographic and discrete morphological differences. Two widespread clades co-occur broadly throughout eastern wet forests, one clade is endemic to western dry forests, and several slightly admixed clades are more narrowly distributed in mountainous regions in the north. Multiple previously described taxa were recovered as paraphyletic in our analyses, some of which were associated with admixed individuals, suggesting that hybridization contributes to taxonomic difficulties in *Canarium*. An improved understanding of *Canarium* species diversity has important implications for conservation efforts and understanding the origins of diversity in Madagascar. Our study shows that even limited genomic sampling, when combined with geography and morphology, can greatly improve estimates of species diversity for difficult tropical clades.

## Introduction

The evolutionary processes underlying the origin and maintenance of species diversity in tropical forests—some of the oldest and most diverse ecosystems on Earth—have long been a source of fascination and debate among biologists [1, 2]. Although tropical forests are known to be species rich, precise estimates of their diversity, and variation across space, are difficult to obtain due to limitations of sampling and our ability to accurately circumscribe species. Refining these estimates is important, however, as it directly impacts a variety of fields that rely on species as units of analysis, including conservation, macroecology, and macroevolution [3, 4]. Here we focus on the issue of species discovery and delimitation. Diversity estimates fluctuate as taxonomic revisions identify new taxa, or existing names are synonymized, and the balance of these two processes can depend on the availability of data and the application of different methods, both of which have changed dramatically in the last decade [5, 6]. In particular, the ease with which massive genetic sequence data can now be obtained and statistically analyzed for species delimitation is relevant. How will estimates of species diversity change as we apply these new methods, especially within relatively poorly studied clades such as tropical trees? [7–10].

Molecular-based species delimitation methods are particularly promising for their potential to overcome sampling problems commonly encountered in the study of tropical trees, where widespread and exhaustive sampling is rarely possible [10]. Many tropical plant species are described from few specimens (for example, see [11]), and the problem of sampling taxonomically informative specimens is especially difficult when reproductive timing is unknown, and when taxa are dioecious. While the importance of increased collecting efforts is clear, molecular-based methods that can be used to infer phylogenetic relationships, and to characterize emergent properties of species, such as population size and divergence time, from few individuals, have the potential to dramatically increase our understanding of diversity using the limited number of specimens that are already available.

It is important, however, that molecular based species delimitation methods continue to be interpreted in light of additional sources of evidence, and biological realism, by integrating results with existing knowledge of the morphology and geographic distribution of species. Without this, patterns of genetic structure alone may lead to inflated estimates of species diversity [12]. At this juncture, there is particular need for detailed case studies to be performed across multiple clades. It is in this spirit that we present the results of our integrative analyses of *Canarium* (Burseraceae), a dominant clade of trees in the tropical forests of Madagascar.

Our aim is to determine the number of independently evolving species in the *Canarium* clade that is endemic to Madagascar. This group is an excellent system for the application of genomic species delimitation methods because two of us (Federman and Daly) recently carried out a morphology-based revision of Malagasy *Canarium* [11]. Additionally, as is the case for many tropical groups, many species of *Canarium* are known from only a few specimens. Our recent taxonomic treatment increased the number of Malagasy species from one (Leenhouts [13]) to 33 (Daly et al. [11]). Although our revision was based on approximately 1,000 herbarium specimens, many lacked reproductive tissues and were therefore of limited taxonomic value. In fact, only seven of our 33 species are known from complete material (i.e. with staminate flowers, carpellate flowers, and fruits). Here, we ask whether genomic data, in combination with morphology and geography, support or revise the number of species recognized in the previous treatment (Daly et al. [11]). We also consider how these findings relate to broader questions of speciation and conservation in tropical trees.

## Materials and methods

### Data collection

**Fig 1:**
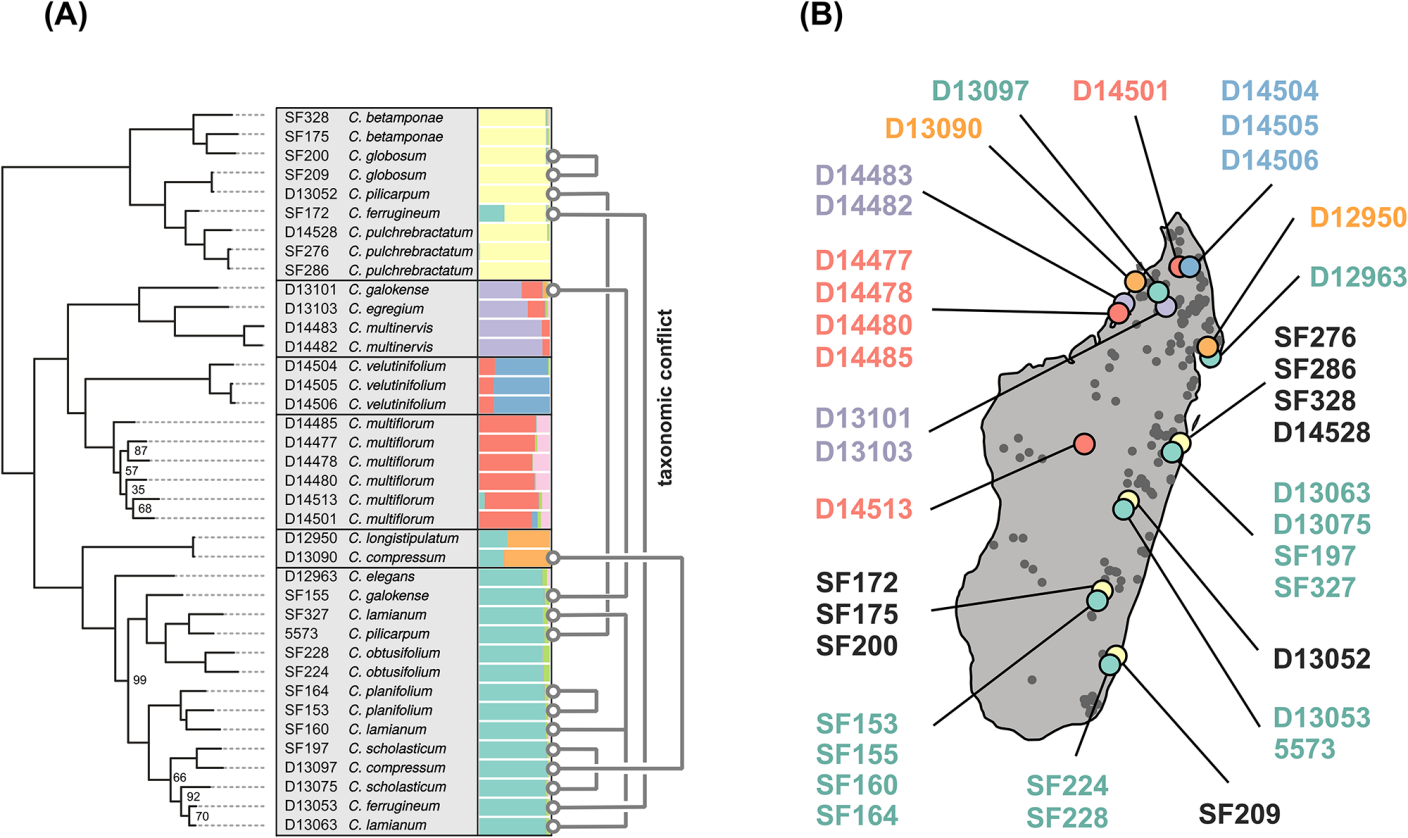
Phylogenetic inference and genetic structure analysis for 38 samples of Malagasy *Canarium*. (A) Maximum likelihood tree inferred from the min20 dataset with bootstrap support values. Accession IDs are shown alongside taxonomic identifications based on [11]. Many names are paraphyletic, as indicated by grey lines connecting labeled samples. A barplot shows the assignment of individual ancestry to eight genetic clusters. (B) A map of Madagascar with 307 georeferenced *Canarium* collections shown as grey points. Accessions included in our genomic analyses are indicated by points colored by their genetic cluster assignment. Points from the same location are offset slightly for visualization purposes.

Many new specimens were collected for this study over the course of five expeditions between 2006 and 2014 that covered the known geographic range of *Canarium* and targeted localities where rare and locally endemic species were previously collected (Fig 1). Specimens were identified using the key of Daly et al. [11], the most inclusive and recent taxonomic treatment, and deposited in herbaria of the Parc Botanique et Zoologique de Tsimbazaza (TAN) and the New York Botanical Garden (NY). For each specimen leaf tissue was stored in silica for molecular analyses, and geographic coordinates and elevation were recorded.

DNA extractions were attempted for all taxa for which recent and sufficient tissues were available. Of the samples that were successfully extracted, at least two individuals for each taxon were included when available. This yielded 44 Malagasy *Canarium* accessions, to which we added an additional four Southeast Asian *Canarium* species to serve as outgroups. Genomic libraries were prepared for genotyping-by-sequencing (GBS) following the protocol described by Escudero et al. [14], but with the addition of a size selection step. After restriction digestion, fragment sizes were visualized on an Agilent 2100 Bioanalyzer and selected in the range of 300–800 bp using a Pippin Prep system. The final library containing 48 barcoded individuals was sequenced on two lanes of an Illumina HiSeq 2500 at Yale University’s Center for Genome Analysis to generate 75 bp single-end reads.

We used ipyrad v.0.7.20 [15] (https://github.com/dereneaton/ipyrad) to assemble sequenced reads *de novo*. Parameters were selected to allow sufficient sequence divergence between samples, while also filtering loci that contain an excess of diversity. The datatype parameter was set to ‘gbs’, common Illumina adapters were trimmed from reads using filter setting ‘2’, and the sequence similarity threshold was set to 90%. The final five bases of each locus were trimmed after alignment to reduce potential alignment errors, and loci with more than 10 SNPs were excluded. We filtered for potential paralogs by allowing heterozygous sites to be shared across a maximum of four samples per locus. All other parameters were left at their default values. The resulting data set included all loci shared across four or more samples, which we refer to as the min4 data set. We assembled additional data sets using more stringent values, to retain loci shared across a minimum of 10 (min10) or 20 (min20) samples, and we generated a smaller data set with a minimum taxon coverage of 30, and outgroups excluded (min30no), for use in population structure analyses.

### Population structure

We used the program STRUCTURE v2.3.4 [16] to cluster individuals into K distinct populations using the admixture model applied to the min30no SNP dataset (17,669 loci), and tested over multiple values for K (2-10). We ran 20 replicates per test, each started from a different random seed, and for each replicate a different subset of SNPs was sampled from the total 64,645 SNPs present in the min30no dataset by randomly sampling a single SNP from each variable locus so that the resulting approximately 17K SNPs are putatively unlinked. Each replicate was run for 500K MCMC steps after a burn-in of 100K. Results were summarized using CLUMPP v.1.1.2 [17]. Convergence of runs was assessed from the variance in log likelihood scores, and replicates were excluded if the variance was greater than 100X the minimum value found among all replicates for that value of K. After removing runs that failed to converge the best fitting value of K was calculated by the Evanno method [18].

### Phylogenetic inference

We employed four approaches to infer phylogenetic relationships: (1) maximum likelihood (ML; [19]); (2) quartet based species tree inference [20]; (3) concordance factor analysis (CFs; [21]); and (4) Bayesian multi-species coalescent (MSC; [22]). The latter two approaches are difficult to apply to large multi-locus data sets, so for these we subsampled data based on results of the two former analyses.

An ML tree was inferred with RAxML v.8.2.10 [23] for each data set (min4, min10, and min20) from the full concatenated sequence alignment under the GTRGAMMA nucleotide substitution model. We used the “-f a” algorithm to perform a full search for the best scoring ML tree after performing 100 rapid non-parametric bootstrap replicates. A quartet based species tree was inferred using tetrad v.0.7.20 [24]. This method is well suited for GBS data since it maximizes the amount of information that can inform each possible quartet regardless of missing data among other samples. Tetrad was run on the largest assembled data set (min4) to include 593,832 SNPs, and infer all 111,930 possible quartets, which were subsequently joined into a supertree with QMC [25]. To assess support we ran 100 non-parametric bootstrap replicates in which loci are resampled with replacement, and in each replicate, a single SNP from each variable locus is randomly sampled to yield new alignments of putatively unlinked SNPs.

To assess the extent of gene tree discordance underlying species relationships we used concordance factor analysis in BUCKy v.1.4.4 [26]. This method summarizes a posterior set of gene trees estimated for multiple loci under a Bayesian framework that accounts for gene tree estimation error. We included a single individual to represent each of the ten major clades recovered in ML analyses by sampling the taxon in each clade with the most recovered GBS data. For this analysis we used only loci for which data was recovered across all included samples, and for which at least two parsimony informative SNPs were present, yielding 750 loci. Gene tree posterior distributions were estimated for each locus in MrBayes v.3.2.2 [27], running two MCMC chains, each for 4M generations following a 1M generation burn-in, and sampling 2000 trees from the posterior for concordance analysis. We analyzed the posterior gene tree distributions in BUCKy in four replicate analyses with four MCMC chains, each run for 1M generations following a ten percent burn-in. The primary concordance tree topology was constructed of clades with the highest non-conflicting CFs, and significance of clade CFs was assessed by overlap in their 95% confidence intervals.

We fit data to the multi-species coalescent model in the program BPP v.3.3 [22] using a fixed topology (algorithm “00”) and also tested alternative species delimitation hypotheses (algorithm “01”). Both analyses used the primary concordance tree topology estimated from BUCKy but with outgroups excluded. We used phased allele data from the ipyrad assembly and included only loci for which at least four individuals had data for each taxon, except for clades with fewer individuals, for which all were required to have data. Individuals with evidence of admixture were not selected for this analysis. To assess the influence of priors on our results we ran analyses under a combination of values for the priors on *θ* ((2, 200) and (2, 2000)) and *τ* ((2, 200) and (2, 2000)). We ran five replicates for each analysis from a different starting seed, and for computational reasons limited each analysis to 100 randomly sampled loci, where each replicate sampled a different random set. Each test was run for 100,000 steps with a burn-in of 10,000 using a sample frequency of 50. Estimated *θ* and *τ* parameters were converted to effective population size and geological time estimates respectively, using the procedure of Yoder et al. [28]. For this we set a prior on generation time to be gamma distributed with a 95% confidence interval (CI) between 8-16 years, and used a gamma prior on mutation rate with a 95% CI between 5e-7 and 5e-8.

### Admixture inference

To test for admixture between lineages we calculated D-statistics (i.e., ABBA-BABA tests; [29]), which quantify asymmetry in the distribution of SNP patterns that are discordant with a hypothesized species tree topology. To reduce the total number of tests, and to assess evidence of admixture between lineages, rather than between individuals, we calculated D-statistics using SNP frequencies for pooled sets of individuals in each species hypothesis [29]. Test significance was measured by performing 1000 bootstrap replicates of each test in which loci are resampled with replacement [30]. Results are reported as test statistics (Z), representing the number of bootstrap standard deviations the D-statistics deviate from zero.

### Reproducibility

Sequence data were deposited in the NCBI sequence read archive under BioProject accession SRP106882. Fully reproducible code is available in the form of jupyter notebooks (https://jupyter.org) with instructions to install all necessary software, download sequence data, assemble it, and run genomic analyses (https://github.com/dereneaton/Canarium-GBS) (DOI 10.5281/zenodo.1273357). For all genomic analyses in this study we used the ipyrad Python API and the ipyrad-analysis toolkit (https://github.com/dereneaton/ipyrad) which provides a set of wrappers around common genomic analysis tools to run highly parallelized analyses using simple Python scripts.

### Integrating morphological, climatic, and molecular data

We examined species morphological and climatic distributions to visualize their concordance with different phylogenetic hypotheses. Morphological data was measured for 11 continuously-varying vegetative characters and three discrete traits (S3 Table). To account for intra-specific and intra-individual variation in continuous characters measurements were recorded as ratios (e.g. the ratio of leaf length to petiole length) and averaged across three leaves for at least five individuals of each species (except *C. egregium* and *C. galokense* which had fewer than 5 individuals and for which all available collections were used).

Continuous variables were analysed with linear discriminant analysis (LDA) in the ‘MASS’ package [31] in R [32] to examine the overlap of species traits in morphospace under several hypotheses for the number of distinct species. Confidence intervals were estimated by training set re-substitution evaluation with jackknifing [31]. The phylogenetic distribution of three discrete traits was also visualized under different species delimitation hypotheses to examine how polymorphic traits segregate within and among clades. The three discrete traits were used previously in the 33-species revision by Daly et al. [11], where they are described in detail, and include: (1) fruit color, (2) pubescence on the underside of leaflets, and (3) stamen position in male flowers.

To explore the biogeographic implications of our species hypotheses, we compiled 307 geo-referenced *Canarium* localities from herbarium specimens of the 17 species recognized by Daly et al. [11] included in our molecular analyses. We used those locality data to quantify niche breadth using the Outlying Mean Index (OMI) ordination technique [33], which is appropriate when sample sizes are small. We ran OMI analyses using the 19 bioclimatic variables from the WorldClim database [34], and used the values from the first ordination axis for ancestral state reconstructions of climatic tolerances using maximum likelihood in the R package ‘phytools’ [35]. We performed OMI analyses and ancestral state reconstructions with samples grouped according to each of the species hypotheses under consideration.

Finally, because fruit size variation in Malagasy *Canarium* has been shown to have important implications for conservation given that it is a major food source for lemurs [4], we also analyzed log-transformed *Canarium* fruit length and width data collected by Federman et al. [4] in relation to a six species hypothesis. We inferred the evolutionary history of fruit size with maximum likelihood using the R package “ape” [36] and projected our phylogeny into a two-dimensional morphological space (following [4]) to consider *Canarium* fruit sizes in relation to the maximum ingestible food size of their dispersal agents: living and extinct lemur species.

## Results

### Specimen collection

We collected 20 of the 33 *Canarium* species described by Daly et al. [11] for inclusion in the GBS library (61% of the estimated diversity; Fig 1; S1 Table), but were unable to incorporate the remaining 13 species which were either known only from herbarium collections that are >20 years old, or had been stored in alcohol which prevented successful DNA extractions. Sampling was highly limited by the fact that 20/33 species are currently known from fewer than 10 specimens. With the exception of *C. bullatum* (known from 21 specimens) we located all species known from more than five collections, and three species known from fewer specimens (S2 Table).

### Genomic assemblies

Our genomic libraries were sequenced to high depth (total 365M reads) yielding an average of 7.6M reads per sample, but with highly variable coverage (stdev = 7.4M), such that several samples had to be excluded for insufficient data. This was likely caused by poor quality DNA extractions, or secondary compounds in them, that may have prevented successful restriction digestion. All samples were assembled to the step where consensus sequences are called (mean = 152,282; stdev = 91,648), at which point samples with fewer than 12,000 consensus sequences were excluded so that the remaining samples would have relatively equal quality and coverage. This resulted in six in-group samples being removed, which excluded three taxa from being represented in downstream analyses. The final assemblies contained 38 in-group samples representing 17 species from Daly et al. [11].

The largest assembly (min4), which allows the most missing data among samples, contains 154,434 loci (67% missing sites) while the min10 and min20 datasets have 81,608 loci (48% missing) and 46,617 loci (33% missing), respectively (S1 Table). The largest concatenated alignment (min4) is 42 taxa x 9.47 Mbp (593,832 SNPs), while the smallest (min20) is 2.8 Mbp of sequence data (245,796 SNPs). All maximum likelihood analyses yielded similar trees that were identical across the backbone of the topology, with only slight differences in topology and support values among several tip-level relationships (Fig 1). The quartet-based species tree analysis yielded a highly similar topology to the ML trees but with lower support values for several splits (S1 Fig). Consistent across all of our phylogenetic analyses, only 5/11 of the species recognized by Daly et al. [11] for which we had multiple individuals sampled were recovered as monophyletic (Fig 1; *C. pulchebracteatum, C. multinervis, C. velutinifolium, C. multiflorum*, and *C. obtusifolium*).

### Phylogenetic and admixture analyses

Three major clades were consistently recovered across all of our phylogenetic analyses, which we refer to as clades 1-3, and which correspond geographically and morphologically to three subspecies circumscribed in Leenhouts’ [13] taxonomic treatment. Clades 1 and 3 both include eastern wet forest adapted trees that Leenhouts circumscribed as *C. madagascariense* Engl. subsp. *bullatum* Leenh., and *C. madagascariense* Engl. subsp. *obtusifolium* Scott Elliot, respectively. The other lineage, clade 2, corresponds to *C. madagascariense* Engl. subsp. *madagascariense*, a wide-ranging lineage found in western dry-forests.

Population structure analyses clustered individuals into the same three clades, but also uncovered finer structure within each, corresponding to further resolved phylogenetic relationships and admixture. The best fitting model clusters individuals into five distinct genetic clusters (Fig 1), followed by models with 4, 3, or 8 clusters, respectively (S2 Fig; S4 Table). Many of our structure runs initially failed to converge for higher values of K when the burnin and MCMC chain lengths were run for shorter periods, which required us to increase these values to 100K and 500K, respectively. This ensured that at least twenty replicate runs converged based on our criteria for all tested values of K. The recovered genetic clusters are concordant with clades recovered in phylogenetic analyses (Fig 1) and also show evidence of admixture between clades.

**Fig 2:**
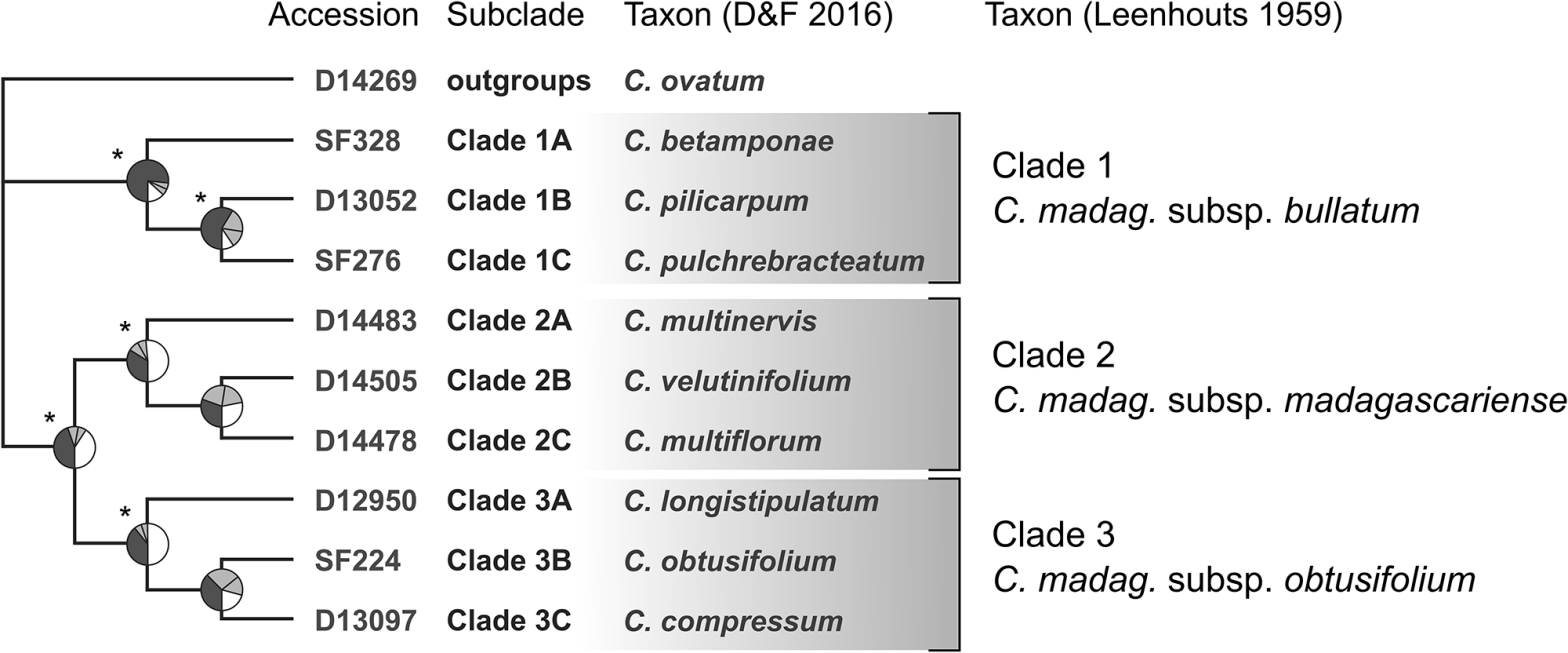
Primary concordance tree for ten clades of *Canarium*. Pie charts indicate concordance factors (CFs) for each split in the tree relative to conflicting splits from alternative trees. The CF for the clade in the tree is shown in dark grey, CFs for the two best supported conflicting clades are in light gray, and the summed CFs of all other conflicting clades are in white. An asterisk indicates significant support for a split, meaning the 95% confidence interval for its CF does not overlap with any conflicting clade. Taxonomic associations from [11] and [13] are indicated for each named subclade.

The primary concordance tree matched the ML and quartet based tree topologies for the ten samples included in this analysis (Fig 2). Concordance factors provide a meaningful interpretation of support by revealing the relative proportion by which different discordant relationships are observed among sampled genes. Five of the seven clades in the primary concordance tree are significantly supported (do not have overlapping 95% confidence intervals with any conflicting clade) while the remaining two show significant conflict for alternative relationships among the three closest subclades within clades 2 and 3 (Fig 2). We will refer to this subclade structure in subsequent tests using a designation of three subclades (A, B, and C) within each clade. In addition, we will refer to joint clades, like 2BC, as containing both clades 2B and 2C.

### Species delimitation

Species delimitation results from BPP were highly consistent across replicate runs that sampled different subsets of loci, as well as across tests using different priors. Because all results agreed very closely we will present only those for the prior setting (*τ* = (2, 2000), Θ = (2,2000)), summarized across five replicate runs. Convergence of individual runs was assessed from ESS values for analyses run under the 00 algorithm, where all parameters showed convergence with ESS > 200. The species delimitation algorithm works by iteratively collapsing or uncollapsing nodes of a tree to join together subclades. In our results, the nine species hypothesis—where all subclades of the three major clades are resolved—received the highest posterior probability (PP = 0.90; S5 Table). This was followed by much lower, but nonzero, posterior probabilities for hypotheses with eight (PP = 0.08) or seven species (PP = 0.02), respectively. Collapsed subclades were only observed in the posterior results for clade 1, where either 1BC was collapsed, or 1ABC was collapsed.

We also fit the multi-species coalescent model to a species tree with all nine subclades using BPP algorithm 00 and converted the estimated parameters to geological time and effective population size (Table 1). These values were calculated from a combined posterior of 300,000 MCMC samples across replicate runs. The crown age for all Malagasy *Canarium* was estimated at 3.09 Mya (95% CI: 1.5-5.64), which is within the 95% confidence interval of previous estimates from a fossil-calibrated Sanger sequence phylogeny [37]. Each of the three major clades diverged >2 Mya, and only the youngest clades (splits within clade 1 and clade 3BC) have estimated divergence times <2 Mya.

**Table 1:** Parameters of the multi-species coalescent model fit by BPP for nine subclades of *Canarium*.

Type	Parameter	Mean	(95% CI)
Tau	1B,1C	1.02	0.25–2.34
Tau	1A,1BC	1.56	0.63–3.21
Tau	2B,2C	2.59	1.29–4.76
Tau	2A,2BC	2.86	1.45–5.18
Tau	3B,3C	1.54	0.66–3.06
Tau	3A,3BC	2.01	0.90–3.96
Tau	2,3	2.86	1.46–5.18
Tau	1,23	3.09	1.56–5.64
Ne	1A	63.69	27.10–127.54
Ne	1B	27.30	9.61–59.42
Ne	1C	43.51	13.29–99.21
Ne	2A	82.79	38.94–157.63
Ne	2B	47.50	19.32–96.75
Ne	2C	260.30	135.99–467.66
Ne	3A	28.59	11.25–61.46
Ne	3B	140.52	66.37–264.61
Ne	3C	110.46	52.93–213.05

Estimated age of clades (Tau) is reported in millions of years, and effective population size (Ne) is reported in thousands of individuals for each terminal clade in the tree.

The clades with the largest estimated effective populations, 2C, 3B and 3C are also the most geographically widespread (Table 1). Although clade 1 is also geographically widespread, its estimated subclade population sizes are much smaller. The lack of differentiation in estimated population sizes among these subclades is consistent with there being less divergence among them. By contrast, subclades within clades 2 and 3 have deeper divergence times and more variable population sizes.

### Introgression

**Fig 3:**
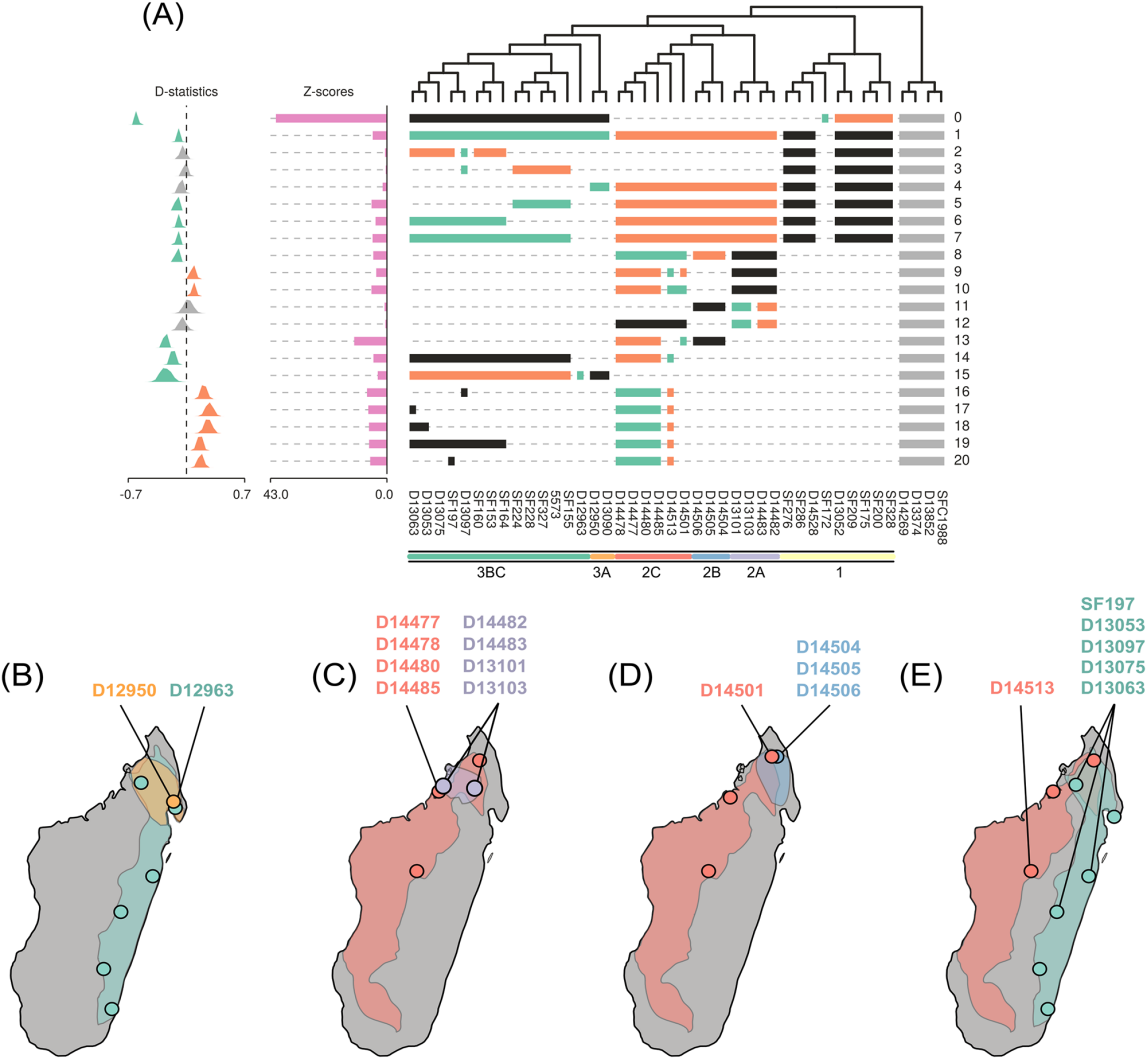
Non-parametric D-statistic tests for admixture. Tests are referred to in the text by their index number shown on the right. Each test compares four clades in the format (((p1, p2), p3), p4);. (A) Horizontal colored bars below the tips of the tree indicate which accessions were included in each test. Bars spanning multiple accessions indicate the use of pooled SNP frequencies to represent a clade. Tests are set up to ask whether the P3 lineage (black bars) shares more derived SNPs with lineage P1 (green bars) relative to P2 (orange bars). Southeast Asian outgroup species were used as the outgroup for all tests (light gray bars). Test significance (Z-scores) is illustrated in a bar plot to the left of each test, and the distribution of D-statistics across bootstrap replicates is shown as a histogram. The histograms are colored to indicate significance when D deviates more than 3.5 standard deviations from zero and to indicate the taxa that are admixed (e.g. the histogram is green for BABA: inferred gene flow between P1 and P3; is orange for ABBA: inferred gene flow between P2 and P3; and gray for non-significant tests). Colored bars at the bottom of the figure are labeled by clade names and correspond to genetic clusters from Fig 1. (B-E) Estimated range maps are shown for admixed taxa to highlight accessions from sympatric versus allopatric populations that show geographically structured patterns of admixture.

We measured D-statistics on a range of topological tests to examine introgression between lineages, and discuss here the most relevant results. We refer to these tests in numbered order as they are shown mapped onto a topology in Fig 3, and for which full statistics are listed in the same order in S6 Table. All tests included the non-Malagasy *Canarium* samples as outgroups. The average number of loci shared across taxa in each test was 35,375, yielding an average of 1,181 discordant ABBA or BABA sites for each comparison.

The most clear signal of admixture is in accession SF172, which appeared highly admixed between clades 1 and 3 in our structure analyses, and which ABBA-BABA tests confirm is a hybrid sharing many more alleles with clade 3 compared to other taxa in clade 1 (D = -0.58; Z = 41.25; Fig 3; test 0). Because this sample is likely an F1 hybrid, or recent back-cross, we exclude it from all further tests when referring to clade 1.

**Fig 4:**
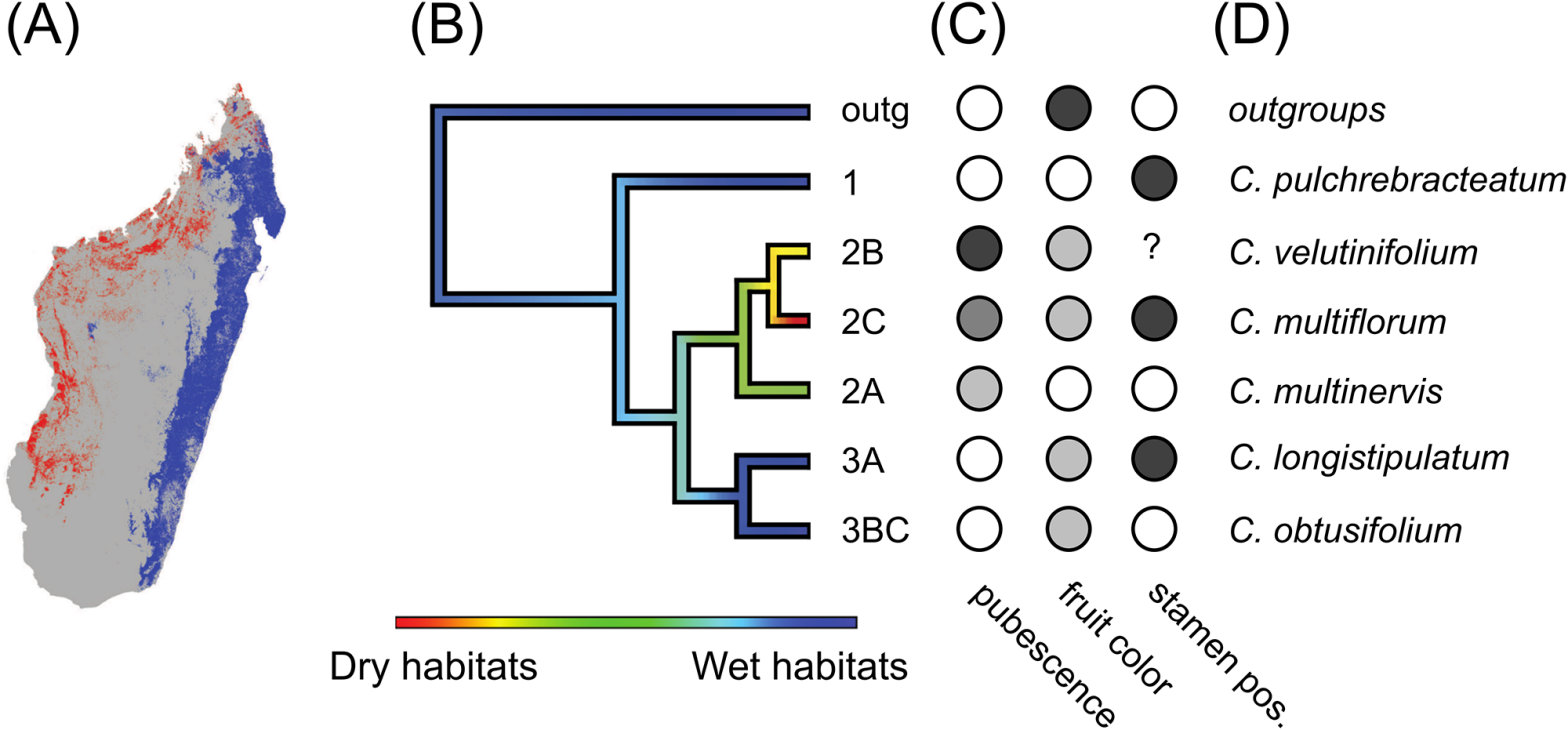
Mapping of discrete traits and climatic variables onto a six species tree of Malagasy *Canarium*. (A) Suitable dry- and wet-forest habitat for *Canarium* based on remote sensing data from the Atlas of Madagascar (Royal Botanic Garden, Kew; http://www.vegmad.org). (B) Phylogeny for the six clades favored under our integrative species delimitation analyses. Colored edges on the tree show ancestral reconstructions of climatic preferences based on the first axis from outlying mean index niche separation analyses. (C) Discrete morphological traits are shown with filled circles for (1) leaf pubescence (from light to dark = glabrous, flexuous hairs evenly distributed, erect hairs in tufts, erect hairs distributed evenly); (2) fruit color (from light to dark: maturing green, maturing brown, maturing purple); and (3) stamen position (white = inserted at disk base, black = inserted outside of disc, ? = unknown). (D) Taxonomic labels associated to the six clades supported under our integrative species delimitation approach.

We next looked for a more diffuse signal of admixture between clades 1 and 3, relative to clade 2, as might be expected if introgression has occurred between them throughout eastern wet forests where they co-occur broadly. Here, we detect a relatively weak signal of admixture (D = -0.07; Z = 5.21; Fig 3; test 1) which appears limited to clades 3B and 3C (D = -0.08, Z = 5.71 and D = -0.07, Z = 4.21 respectively; tests 5-7), since clade 3A shows no such pattern (D = -0.04, Z = 1.59; test 4). The absence of admixture from clade 1 into clade 3A makes sense considering the geographic distribution of clade 3A which occurs in the northern mountainous regions mostly outside the range of clade 1. This area north of 16 degrees south has greater interdigitation of wet and dry forests (Fig 4A) and has therefore been hypothesized to be an area of species generation for other organisms [7]. One exception to this, however, is sample D13097 from clade 3, which despite being found in the north shows similar levels of admixture with clade 1 as other samples in clade 3BC (tests 2-3), suggesting it expanded into this area after introgression occurred into this clade.

In general, the northern region of Madagascar harbors the greatest diversity of *Canarium* lineages, and these lineages are the most admixed. This includes several clades endemic to this region, such as 2A, 2B, and 3A, as well as several widespread lineages whose ranges extend into this region, like 2C, 1, and 3BC. These widespread lineages provide particular power to our analyses for detecting admixture by allowing us to test for differences in sympatry versus allopatry (e.g., [38]). For example, the accession D12963 from clade 3BC was sampled in the northern extent of its clade’s range, where it occurs in sympatry with clade 3A, and we find that this sample shares many more alleles with clade 3A than any other sample in clade 3BC (D = -0.22, Z = 3.39; test 15; Fig 3B). The admixed ancestry of sample D12963 may explain why its phylogenetic position was variable within clade 3BC across phylogenetic analyses (S1 Fig).

A similar geographically structured signal of introgression is also apparent for several subclades in clade 2 (Fig 3C–3E). For example, clade 2A is admixed with clade 2C relative to clade 2B (test 8), and when we compare different samples from within the widespread clade 2C we find that those from the same location as clade 2A share significantly more alleles with it than those from allopatric regions (Fig 3C; tests 9-10). We do not find significant variation between the two sampled locations for clade 2A in their admixture proportions (tests 11-12) suggesting that this lineage has likely been more of an introgressive donor than recipient with clade 2C.

Among populations of the widespread taxon 2C we find additional examples of localized admixture. In the far north it co-occurs with another endemic taxon, clade 2B, where we again find the strongest signal of admixture among sympatric accessions relative to allopatric accessions (Fig 3D; test 13). Similarly, sample D14513, our southernmost accession of the widespread taxon 2C, shares significantly more alleles with the widespread clade 3 (test 14) and in particular, with samples from clade 3C that were sampled nearest to it (test 16-20; Fig 3E). This result is surprising since today these clades occur on separate sides of the central plateau. However, if in the past more expansive forests once spanned the central plateau, connecting these populations, then admixture could have occurred between them in central Madagascar. Similar evidence for a connection across the central plateau has been described previously in mouse lemurs [28].

### Morphology and environment

To interpret the results of our genomic analyses, which found support for 7-9 distinct genetic clades, we now turn to examine the identity of these clades in terms of the taxonomic names that were assigned to individuals in them, and the morphological, geographic and climatic variation they represent. For taxa that could not be easily mapped from the Daly et al. [11] treatment to our hypotheses for 3-9 species, due to paraphyly of the larger set of names, individuals were assigned to the following clades based on our best predictions (*C. pilicarpum* = 3B, *C. galokense* = 3B, *C. compressum* = 3C, *C. ferrugineum = 3C*).

As a conservative estimate of species diversity, and to ensure that morphological data is present for all traits for each clade, and that at least some consistent discrete morphological difference can be identified, we collapsed the nine clades we have been investigating into six, or fewer, for further morphological analyses. This made sense since some clades, like 1 and 3BC, lacked any discrete morphological variation among the subclades within them. When discrete morphological traits are mapped onto the six clade phylogeny some clades still lacked sufficient data to map all traits (e.g., clade 2B lacks stamen position information). Still, from the data available, we find that some traits exhibit phylogenetic signal (e.g., leaf pubescence), while others exhibit homoplasy (e.g., mature fruit color, stamen position; Fig 4C), which has likely contributed to taxonomic difficulties in *Canarium*.

Linear discriminant analyses of 12 morphological traits across 185 leaves from 85 specimens showed differentiation into recognizable clusters (with some overlap) when specimens were grouped into hypotheses for three (1, 2, 3), four (1, 2, 3A, 3BC), five (1, 2A, 2BC, 3A, 3BC), or six species (1, 2A, 2B, 2C, 3A, 3BC). When only three species are recognized (clades 1, 2, and 3), clade 3 is polymorphic for stamen position, and clade 2 is polymorphic for all three characters. When four species are recognized, clade 3 is split into 3A and 3BC, and these become monomorphic for stamen position. With the recognition of five species—distinguishing 2A from 2BC—each species is monomorphic for stamen position and fruit color (Fig 4C). However, clade 2BC remains variable with respect to pubescence: some individuals have leaflets completely covered in dense flexuous hairs, while others are glabrous except for tufts of hairs in the axils of the secondary veins. When 2BC is split into 2B and 2C, each subclade becomes uniform with respect to pubescence. The leaflets in 2C have tufts of hairs only, while those of 2B are densely covered in hairs.

Outlying mean index (OMI) analyses for climatic tolerances were consistent across species hypotheses. For the three, four, five and six species hypotheses, variation was largely described by the first axis, which represents a gradient from wet to dry habitats (S7 Table). Ancestral climate reconstructions for all species hypotheses infer a largely wet-adapted ancestor, consistent with the hypothesis that *Canarium* colonized Madagascar from a Southeast Asian clade adapted to rainforest conditions (Fig 4B). Under this scenario, *Canarium* occupied drier forests only later.

Federman et al. [4] inferred that six of the species within the 33-species hypothesis of Daly et al. [11] have fruit too large to be consumed by any living Malagasy primate, potentially jeopardizing their long-term survival. However, if only six species are recognized, the range of variation in fruit sizes within species allows for at least some of the fruits of each of the six species to be consumed and dispersed by the extant but critically endangered lemur species *Varecia rubra* and *V. variegata* (S4 Fig). Thus a revised taxonomic treatment of *Canarium* provides a more optimistic view on the future dispersal and survival of these lineages.

### Taxonomic revision

Our genomic analyses found support for up to nine distinct clades of Malagasy *Canarium*, although support varied across different analyses. Concordance factor analysis did not fully support a split between clades 3B and 3C, and Bayesian species delimitation analyses found lowest support for splits within clade 1. Disagreement among these analyses is not entirely unexpected as each has different underlying assumptions. Concordance factor analysis measures support for clades regardless of the source of discordance, such as introgression, whereas the species delimitation analyses under the multi-species coalescent model assume that discordance is caused by incomplete lineage sorting. Similarly, these analyses differed in the size and identity of the dataset that was used, where BUCKy required subsampling to include only one accession per taxon, while BPP was able to utilize information for multiple accessions per taxon, but was limited to many fewer loci for computational reasons.

Based on all evidence presented, we estimate that there are at least six distinct species of Malagasy *Canarium*, though we can not yet rule out the possibility that some divergent lineages remain to be sampled. For the six species we have delimited here, we provide a new identification key (S1 Appendix) where we have assigned taxonomic names to these clades based on their correspondence to type specimens from the existing treatments (Fig 4D).

## Discussion

We investigated the radiation of *Canarium* species using the framework of a unified species concept [39] in which a consilience of evidence is brought to bear on the question of species delimitation. Our study follows on previous taxonomic studies for Malagasy *Canarium* that have described from one to 33 species [11, 13]. By incorporating genomic data for 17 of the 33 species recognized in Daly et al. [11] we find significant divergence between many subclades supporting the existence of more than one species. While we do not have sufficient sampling to test the validity of all 33 species, our finding that more than half of species with multiple individuals sampled were resolved as paraphyletic suggests that many of these taxa are not distinct. Reciprocal monophyly of sampled accessions is not the sole basis for this conclusion, however, but rather our finding that taxonomic conflict was most widespread in clades 1 and 3, and that these clades showed the least genetic divergence among subclades or morphological differentiation. Results like ours, in which morphological and molecular estimates of species diversity and relationships differ, may not be uncommon for dioecious tropical tree clades like *Canarium* where many taxa are described from few specimens that often lack full reproductive tissue from both male and female plants.

Statistical species delimitation approaches are intended to remove subjectivity from the assignment of species status to clades or subclades of varying degrees of divergence. Yet, it can be argued that the evolution of incompatibilities, or other features that distinguish species, do not necessarily coincide strongly with coalescent times, but can be idiosyncratic in their origin. In our delimitation of *Canarium* species we take into account species delimitation results from BPP, but retain some subjectivity by incorporating our knowledge of *Canarium* geography, environment, and morphology.

Although limited sampling prevents us from testing the validity of all described taxa in *Canarium*, our findings indicate that the radiation of *Canarium* in Madagascar has been less extensive than previously proposed. Even if all of the rare taxa not present in our dataset turn out to be highly distinct genetic lineages, there would still be a reduction in the number of species from 33 to 22, since we believe our analyses have refuted many taxa by showing evidence of extensive paraphyly. Nevertheless, our hypothesized number of *Canarium* species still represents a significant increase from the one species or three subspecies proposed by Leenhouts [13]. This highlights the importance of a clade-by-clade approach to diversity estimates, as well as the need for increased support for targeted collection efforts of rare, phenotypically distinct, and incompletely known taxa.

### Species delimitation and evolution in Malagasy *Canarium*

The major clades supported by our phylogenetic analyses correspond to six well supported genetic clusters in Structure (Fig 1A) that are distinguishable by continuously varying morphological traits (S3 Fig) and by discrete morphological differences and climatic tolerances (Fig 4B and 4C), and are estimated to be several million years diverged (Table 1). Moreover, we find consistent evidence of limited admixture between sympatric lineages in almost every region they co-occur, yet no indication that extensive hybridization is likely to breakdown barriers between species, as admixture proportions were always very low except in a single accession that was likely a direct hybrid. This result, showing that lineages can co-occur broadly yet retain distinct genetic and morphological differences, is perhaps the strongest evidence in support of recognizing these lineages as distinct species.

Based on the six-species hypothesis, we envision a wet-forest adapted species arriving in Madagascar from Southeast Asia with little to no pubescence, and with fruits that mature from green to purple, reflecting the bird dispersal syndrome in Asian relatives [37]. In connection with a switch to lemur dispersal in Madagascar, the purple phase of fruit development may have been lost as an adaptation through delayed development so that fruits remain green at maturity, and brown fruits would have evolved twice from this ancestral condition. Under this scenario, *Canarium* shifted later into drier forests and evolved more coriaceous and pubescent leaflets.

It remains possible that additional species of Malagasy *Canarium* will be recognized, or currently unsampled species will be validated, as collecting efforts and analyses expand in the years to come. However, based on our morphological studies [11], we predict that the taxa we were unable to include here would broadly fall within the range of morphological variation represented by the included specimens, i.e., with the possible exception of *C. bullatum*, they do not display highly distinctive traits that would suggest their placement outside of the major clades identified here.

### Speciation and conservation in Madagascar

An improved understanding of the radiation of *Canarium* in Madagascar bears on our understanding of diversification patterns and conservation issues on the island. While many studies have addressed mechanisms of speciation for Malagasy fauna (see review by [7]), there are few analogous studies in plants (but see [40]). Such investigations are especially important for understanding the processes that shaped Madagascar’s distinctive biomes, which are generally defined on the basis of plant communities (i.e., spiny forests, humid forests, grasslands; [7, 41]. Studies of speciation in Malagasy animal clades have focused attention on barriers to gene flow such as rivers, watersheds, and elevational gradients [42–44]. In *Canarium* we do not observe these same barriers; however, consistent with several previous studies [7, 28, 41], we do see diversification primarily along the wet-dry (east-west) climatic axis (Fig 4A).

Although Madagascar’s major biomes are generally marked by sharp borders, wet and dry forests interdigitate extensively north of roughly 16 degrees south [7]. This region has received much attention as an area of high micro-endemism, and some paleoclimatic models suggest that it harbored rainforest refugia during the Pleistocene [45] and potentially played a major role in Madagascar as a center of diversification [7, 43]. Within *Canarium*, this northern region contains the greatest species diversity, environmental tolerances, and range overlap among lineages, and it is the region in which we see the most evidence of admixture (Fig 3). We hypothesize that this area of high topographic and climatic variation acted as a species pump for *Canarium* during periods of aridification, by creating opportunities for both vicariant divergence in wet-forest mountain refugia, and ecogeographic divergence along wet-dry environmental gradients.

The recognition of fewer species of *Canarium* in Madagascar also bears on important conservation issues. In the past few thousand years it is estimated that at least 17 species of large bodied lemurs have gone extinct [46], and many of these taxa were seed dispersers with gape sizes easily capable of ingesting and dispersing the largest *Canarium* fruit [4, 46]. Under the 33 species hypothesis [11], at least six species of *Canarium* have fruits that are too large to be ingested by any living Malagasy primate [4]. However, when these taxa are lumped into six species with more widely variable fruit sizes, this range of variation can accommodate ingestion by Madagascar’s largest extant frugivores (*Varecia* spp.) (S4 Fig). This provides hope that *Canarium*, though solely reliant on a critically endangered dispersal agent, could experience selection for smaller fruit sizes, as [47] observed in Brazil under similar circumstances.

### Towards an integrative species delimitation framework

Accurate diversity estimates are critical for addressing many issues in evolutionary biology and ecology (e.g., diversification rates, community assembly; [2]), as well as for the development of successful conservation and management strategies [3]. The species delimitation framework used here emphasizes the incorporation of multiple lines of evidence (molecular data, morphology, biogeography, climate) to evaluate the extent to which a clade of dominant tropical trees has radiated in Madagascar. The addition of molecular data has shed considerable light on morphological evolution in *Canarium*. Accessions of a number of the previously recognized species (e.g., *C. ferrugineum*, *C. galokense*, *C. pilicarpum*, and *C. compressum*) did not form clades (Fig 1), suggesting parallel evolution of a number of traits used to delimit these species. Returning to the herbarium to closely re-examine the collections used in our molecular analyses helped us to determine which morphological traits corresponded best with our six-lineage hypothesis, and allowed us to develop a more effective identification key (S1 Appendix). This exercise confirmed the placement of these collections in their respective clades, and provided morphological support for our finding that one collection (SF172) likely originated via hybridization between members of clades 1 and 3 (Fig 3). Many of SF172’s morphological traits, such as leaflet size and shape, reticulum, and petiolules are in fact intermediate between species in clades 1 and 3. We additionally posit that much of the taxonomic confusion and molecular non-monophyly could be due to the prevalence of sterile collections in our analyses, or the accidental incorporation of juvenile or sucker-shoot phenotypes in our earlier morphological delimitations. Such information is often not evident in older herbarium collections, further underscoring the importance of increased sampling efforts.

We began by asking whether the incorporation of molecular data with morphological and geographic lines of evidence would likely increase or decrease the number of species recognized in tropical forests, especially for those many groups with limited complete specimens such as dioecious trees. Our study of *Canarium* shows that under some circumstances fewer species will ultimately be recognized, highlighting the importance of critical clade-by-clade approaches and increased collection efforts. Our result may turn out to be uncommon, as there are no doubt many groups where species diversity remains dramatically underestimated. Although the use of molecular data alone can result in spurious species delimitations [12], our approach clearly demonstrates the utility of molecular data in an integrative context. We will need many more case studies, and increased opportunities for the targeted collection of rare species and phenotypes, to more precisely forecast the trajectory of species diversity in the tropics.

### Data accessibility

Demultiplexed raw sequence data is available on NCBI SRA (SRP106882). Climate and morphological data and code to reproduce all analyses are available at http://github.com/dereneaton/Canarium-GBS/ (DOI 10.5281/zenodo.1273357).

## Supporting information

S1 Fig Phylogenetic analyses for different genomic assemblies with different proportions of missing data. All ML analyses returned similar results whereas the quartet based species tree differed slightly with lower bootstrap support values overall.
(PDF)

S2 Fig Structure barplots for the models with K = 2-10 populations. Results are summarized across 20-40 replicates for each value of K after excluding replicate runs that failed to converge.
(PDF)

S3 Fig Morphological disparity among Malagasy *Canarium*. Linear discriminant analysis of eleven vegetative characters from 185 specimens was used to group individuals into clusters for species hypotheses of 3, 4, 5, or 6 species. Differentiation among species is visually apparent for all hypotheses. Linear discriminant scatter plots are colored according to their hypothesized lineage shown in the key to the right.
(PDF)

S4 Fig Seed size, evolution, and the limits of maximum ingestible food size for extinct and extant frugivorous lemurs in relation to six-species of Malagasy *Canarium*. Log-transformed *Canarium* fruit length and width data projected into a two dimensional phylogenetic morphospace. Lineages are coded with letters. Dashed lines indicate the maximum ingestible food size of extant and extinct (marked with a cross, and shown in green) lemur lineages as calculated by [4]. When the Malagasy *Canarium* are circumscribed as six species, the range of fruit size variation allows for all species to be ingested by extant dispersers.
(PDF)

S1 Table Collection information for sampled individuals included in genomic analyses and their associated genomic assembly statistics. (CSV)

S2 Table Number of specimens known for Malagasy *Canarium* and their reproductive completeness. (CSV)

S3 Table Continuous and discrete traits measured on *Canarium* taxa based on identifications from the key of Daly et al. [11]. (CSV)

S4 Table Population structure analyses for K in 2-10 found highest support for five distinct genetic clusters. Between 20-40 replicate analyses were run for each test, only the number of tests passing our convergence criterion were included when calculating the best model using the Evanno Method.
(CSV)

S5 Table Posterior probabilities for 30 different species delimitation models. Each model represents a single nine species topology with ordered nodes either collapsed or intact based on the species delimitation framework of BPP algorithm “01”. Greatest support was found for the completely resolved model that includes all nine subclades examined.
(CSV)

S6 Table ABBA-BABA results for a number of selected tests examining admixture among clades of Malagasy *Canarium*. Test numbers correspond to results in Fig 3. When SNP frequencies across multiple accessions were used to represent a taxon the names are listed within brackets.
(CSV)

S7 Table Results from outlying mean index ordination analysis for determining climatic tolerances in Malagasy *Canarium*. Ordination axes 1 and 2 reported for 19 climatic variables for each of 4 different species hypotheses.
(CSV)

S1 Appendix Key to the Malagasy *Canarium* according to a six-species hypothesis. (PDF)
