# Supplementary figures and images for: Reconciling species diversity in a tropical plant clade (Canarium, Burseraceae)
Source: PLoS One. 2018 Jun 15;13(6):e0198882. doi: 10.1371/journal.pone.0198882 (PMC6003679; doi:10.1371/journal.pone.0198882)

raxml min4

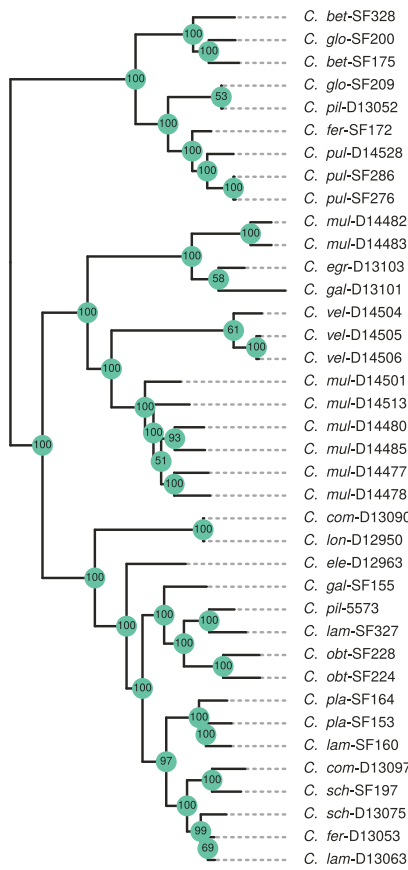

raxml min10

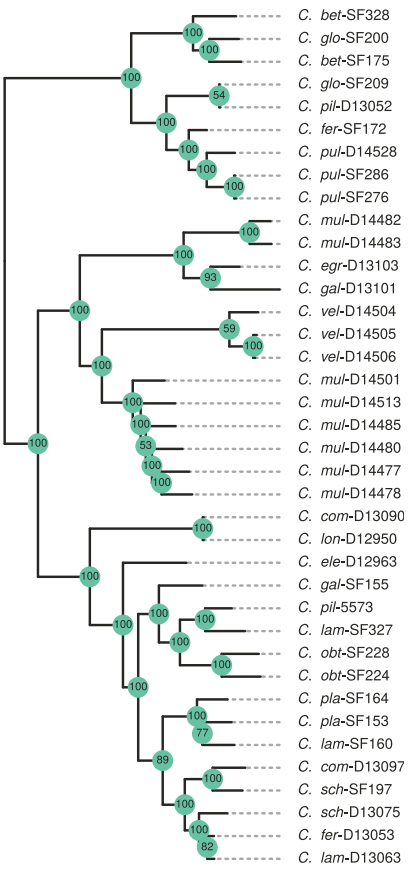

raxml min20

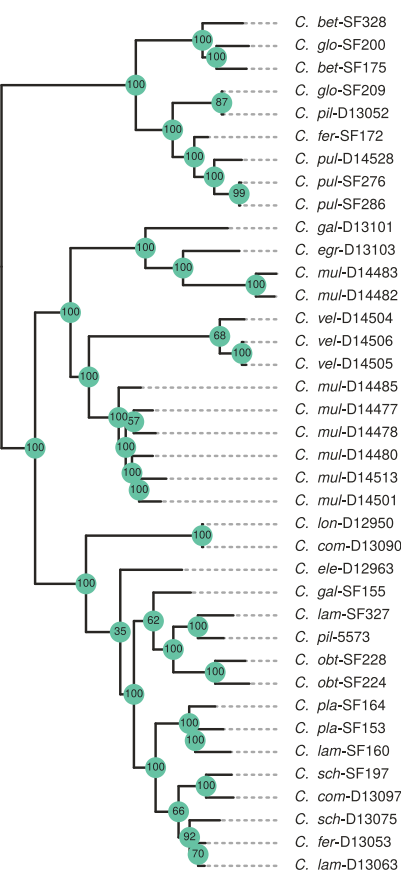

raxml min20  
with outgroups

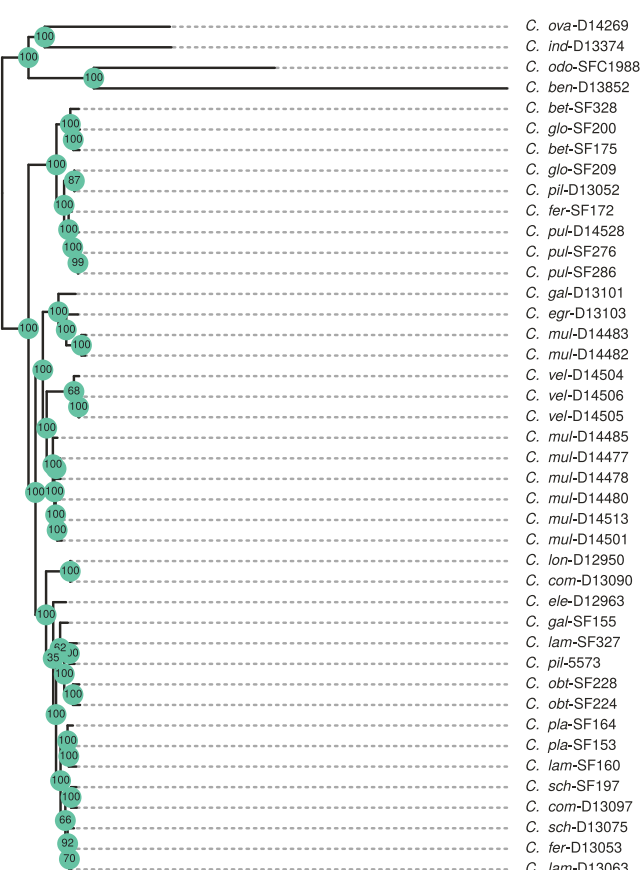

tetrad min4

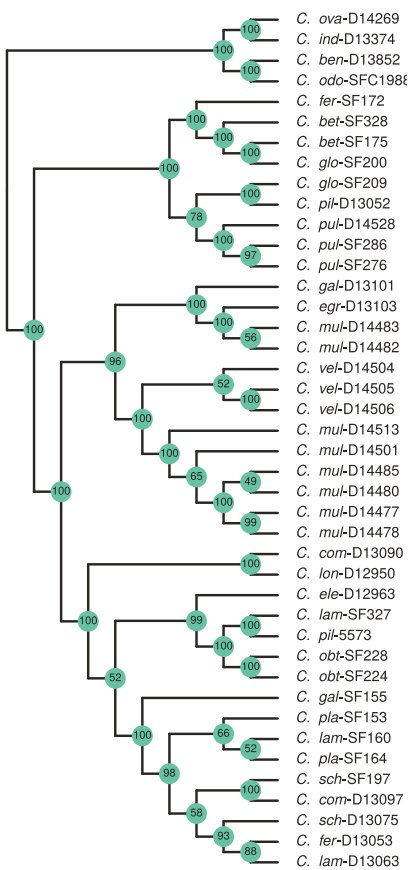

Supplement: S1 Fig — All ML analyses returned similar results whereas the quartet based species tree differed slightly with lower bootstrap support values overall. (PDF) [file pone.0198882.s001.pdf]

K=2 K=3 K=4 K=5 K=6 K=7 K=8 K=9 K=10

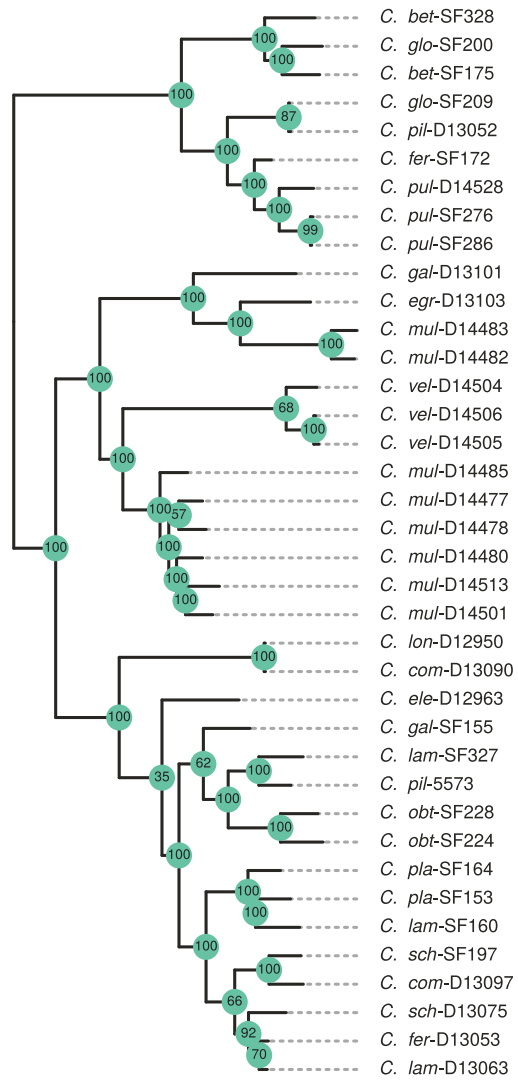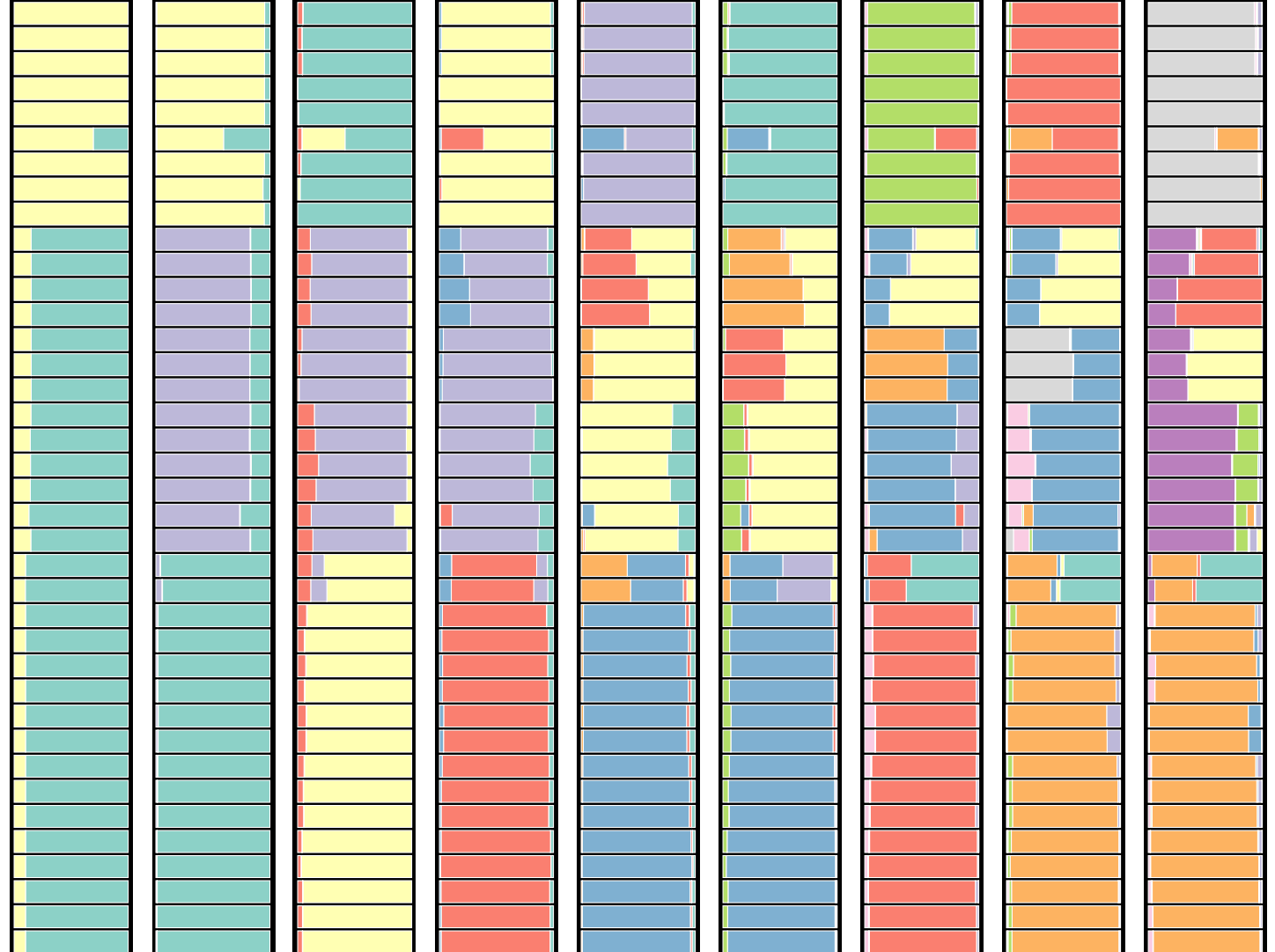

Supplement: S2 Fig — Results are summarized across 20-40 replicates for each value of K after excluding replicate runs that failed to converge. (PDF) [file pone.0198882.s002.pdf]

### 3 species

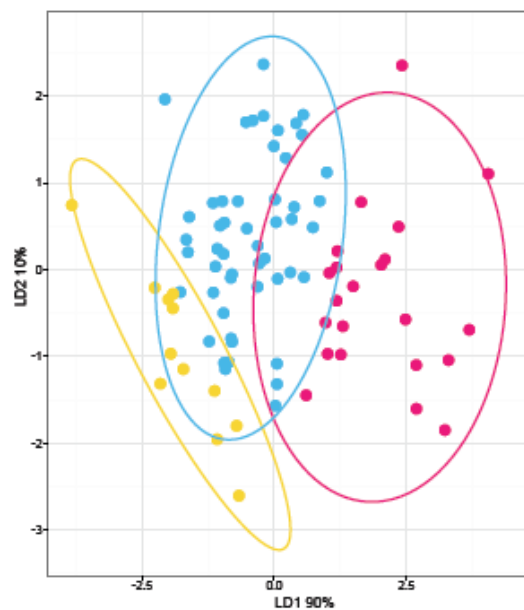

### 4 species

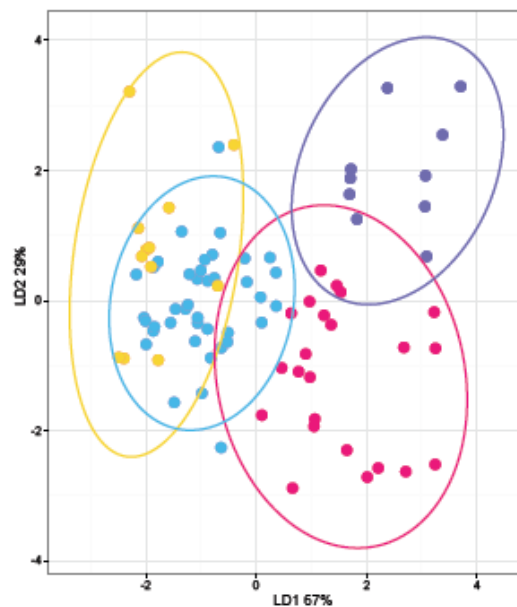

### 5 species

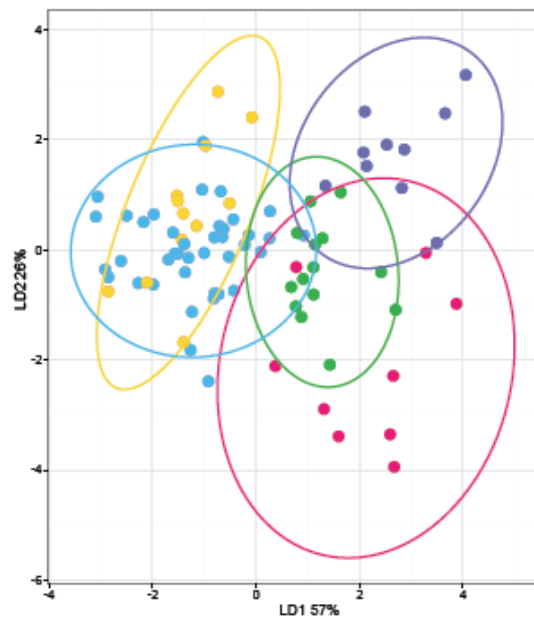

### 6 species

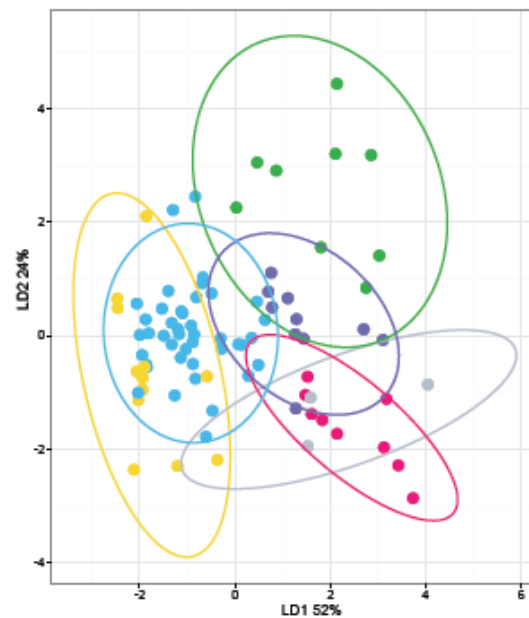

Clade

1

2A

2B

2C

3A

3BC

Supplement: S3 Fig — Linear discriminant analysis of eleven vegetative characters from 185 specimens was used to group individuals into clusters for species hypotheses of 3, 4, 5, or 6 species. Differentiation among species is visually apparent for all hypotheses. Linear discriminant scatter plots are colored according to their hypothesized lineage shown in the key to the right. (PDF) [file pone.0198882.s003.pdf]

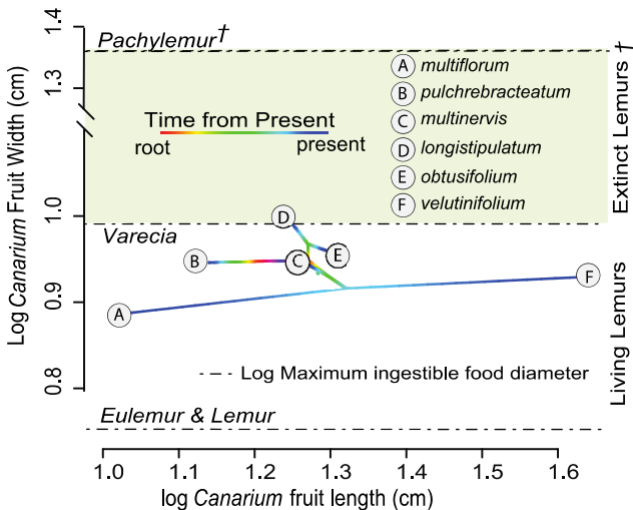

Supplement: S4 Fig — Log-transformed Canarium fruit length and width data projected into a two dimensional phylogenetic morphospace. Lineages are coded with letters. Dashed lines indicate the maximum ingestible food size of extant and extinct (marked with a cross, and shown in green) lemur lineages as calculated by [4]. When the Malagasy Canarium are circumscribed as six species, the range of fruit size variation allows for all species to be ingested by extant dispersers. (PDF) [file pone.0198882.s004.pdf]
