# Supplementary material for: Reconciling species diversity in a tropical plant clade (Canarium, Burseraceae)
Source: PLoS One. 2018 Jun 15;13(6):e0198882. doi: 10.1371/journal.pone.0198882 (PMC6003679; doi:10.1371/journal.pone.0198882)
Supplement: S1 Appendix — (PDF) [file pone.0198882.s012.pdf]

Federman et al. 2018

Canarium L.

Key to the Malagasy *Canarium* according to a six-species hypothesis.

---

1. Leaves 1-5 (rarely 6) jugate; leaflet base acute to truncate (rarely cordate) —— **2**
+ Leaves 5-10 (rarely 4) jugate and leaflet base truncate to cordate (rarely acute) —— **3**

2. Reticulum conspicuous; petiolules usually canaliculate; fruit smooth and green: —— **Clade 1**
+ Reticulum inconspicuous; petiolules rarely shallowly canaliculate; fruit lenticillate and brown: —— **Clade 3A**

3. Leaflets densely pubescent: —— **Clade 2B**
+ Leaflets sparsely pubescent to glabrous —— **4**

4. Petiole densely ferrugineous pubescent; distance of stipule scar from petiole insertion long; fruit lenticillate and brown —— **5**
+ Petiole glabrous to sparsely pubescent; distance of stipule scar from petiole insertion short; fruit lenticillate and green: —— **Clade 2A**

5. Leaflets coriaceous; tufts of hairs in the axils of leaflet secondary veins: —— **Clade 2C**
+ Leaflets chartaceous; tufts of hairs in secondary vein axils absent: —— **Clade 3BC**

Clade 1 (*C. pulchrebracteatum* Guillaumin):

Habit, riverbanks throughout western and northwestern dry forests. Male flowers, stamens inserted outside of the disk at the base; petals slightly spreading and not hooded

Clade 2A (*C. multinervis* Daly, Raharim & Federman):

Habit, locally restricted to northeast and northwest in dryer and more open habitats, usually occurring in mountainous areas. Male flowers, stamens are inserted on periphery and rarely at the base of the disk; petals sub-erect and usually hooded.

Clade 2B (*C. velutinifolium* Daly, Raharim & Federman):

Habit, restricted to northwest and northeast, usually in littoral forests and more open environments.

Clade 2C (*C. multiflorum* Engl.):

Habit, riverbanks throughout western and northwestern dry forests. Male flowers, stamens inserted outside of the disk at the base; petals slightly spreading and not hooded.

Clade 3A (*C. longistipulatum* Daly, Raharim & Federman):

Habit. notheastern moist forests. Male Flowers, stamens usually inserted on the outside of the disk, rarely at the base; petals somewhat hooded; calyx shallowly to deeply cupular sometimes tubular.

Clade 3BC (*C. obtusifolium* Scott-Elliot):

Habit, exposed slopes and ridge-tops throughout eastern and northeastern moist forests. Male flowers, shallow calyx; patent petals; exposed stamens; stamens inserted at base of disk.
